# The Link Between the Ecology of the Prokaryotic Rare Biosphere and Its Biotechnological Potential

**DOI:** 10.3389/fmicb.2020.00231

**Published:** 2020-02-19

**Authors:** Francisco Pascoal, Catarina Magalhães, Rodrigo Costa

**Affiliations:** ^1^Department of Bioengineering, Institute for Bioengineering and Biosciences (iBB), Instituto Superior Técnico, University of Lisbon, Lisbon, Portugal; ^2^Interdisciplinary Centre of Marine and Environmental Research (CIIMAR/CIMAR), University of Porto, Porto, Portugal; ^3^Faculty of Sciences, University of Porto, Porto, Portugal; ^4^School of Science & Engineering, University of Waikato, Hamilton, New Zealand; ^5^Ocean Frontier Institute, Dalhousie University, Halitax, NS, Canada; ^6^Centre of Marine Sciences (CCMAR), University of Algarve, Faro, Portugal; ^7^U.S. Department of Energy Joint Genome Institute, Berkeley, CA, United States; ^8^Environmental Genomics and Systems Biology Division, Lawrence Berkeley National Laboratory, Berkeley, CA, United States

**Keywords:** bioremediation, bioprospection, microbial dark matter, microbial ecology, biotechnology

## Abstract

Current research on the prokaryotic low abundance taxa, the prokaryotic rare biosphere, is growing, leading to a greater understanding of the mechanisms underlying organismal rarity and its relevance in ecology. From this emerging knowledge it is possible to envision innovative approaches in biotechnology applicable to several sectors. Bioremediation and bioprospecting are two of the most promising areas where such approaches could find feasible implementation, involving possible new solutions to the decontamination of polluted sites and to the discovery of novel gene variants and pathways based on the attributes of rare microbial communities. Bioremediation can be improved through the realization that diverse rare species can grow abundant and degrade different pollutants or possibly transfer useful genes. Further, most of the prokaryotic diversity found in virtually all environments belongs in the rare biosphere and remains uncultivatable, suggesting great bioprospecting potential within this vast and understudied genetic pool. This Mini Review argues that knowledge of the ecophysiology of rare prokaryotes can aid the development of future, efficient biotechnology-based processes, products and services. However, this promise may only be fulfilled through improvements in (and optimal blending of) advanced microbial culturing and physiology, metagenomics, genome annotation and editing, and synthetic biology, to name a few areas of relevance. In the future, it will be important to understand how activity profiles relate with abundance, as some rare taxa can remain rare and increase activity, whereas other taxa can grow abundant. The metabolic mechanisms behind those patterns can be useful in designing biotechnological processes.

## Introduction

Low abundance microbial taxa are usually referred to as members of the “Rare Biosphere”(Sogin et al., 2006), a relatively recent, but important concept to understand microbial ecology from a fundamental perspective (Jia et al., 2018). The microbial rare biosphere encompasses a large diversity of prokaryotes and microeukaryotes, both with a recognized role in ecosystem functioning (Logares et al., 2015; Jousset et al., 2017). This Mini Review covers, unless stated otherwise, the recent and larger body of work that unequivocally links the rare status of prokaryotic populations with genotypic traits and activities of potential use in bioremediation and bioprospection. We direct the reader to the reviews by Weisse (2014) and Logares et al. (2015) and the reports by Ziegler et al. (2018) and Murdock and Juniper (2019) for current insights into the ecology of rare microeukaryotes.

In general, the microbial rare biosphere may act as a “seed bank” (Pedrós-Alió, 2006) where cells are dormant or metabolically inactive (Neufeld et al., 2008; Jones and Lennon, 2010), thereby hinting at why they exist in such low numbers. If cells within this seed bank are viable, by changing conditions low abundance microorganisms can become abundant (Shade et al., 2014). Another possibility is that cells in low-abundance populations are metabolically active (Campbell et al., 2011; Hugoni et al., 2013; Logares et al., 2013), but do not grow to become abundant in the environment (Galand et al., 2009; Kirchman et al., 2010; Gobet et al., 2012; Debroas et al., 2015; Liu et al., 2015) likely due to intrinsic metabolic limitations (Pedrós-Alió, 2012). Low-abundance microorganisms can also act as “keystone species,” meaning they have a disproportional effect on specific functions relative to their abundance (Caron and Countway, 2009; Pester et al., 2010; Hausmann et al., 2019). Finally, they can simply be a consequence of random dispersal and exist only transiently (Sogin et al., 2006; Fuhrman, 2009; Jousset et al., 2017). All the above-mentioned observations form the basis for the existence of different “types of rarity” across space and/or time (Vergin et al., 2013; Lynch and Neufeld, 2015; Jia et al., 2018). When DNA-based methods are used to describe the rare microbial biosphere, a considerable proportion of the observed diversity may be representative of dying or dead cells (Pedrós-Alió, 2012).

Independently of the mechanisms explaining rarity and its spatial-temporal behavior, it is now widely accepted that, both within prokaryotes and single-celled eukaryotes (“protists”), the microbial rare biosphere constitutes an important “genomic reservoir” or “pool of diversity” (Youssef et al., 2010; Bowen et al., 2012; Logares et al., 2014, 2015; Lynch and Neufeld, 2015; Fuentes et al., 2016) that is likely to play fundamental roles in ecosystem functioning. Thus, this community of low-abundance species would contain many different genes that could be used for a variety of functions. Besides, the rare biosphere may also perform the same functions of the abundant biosphere, conferring “functional redundancy” to the entire community (Szabó et al., 2007; Coveley et al., 2015). Particularly within prokaryotes, the metabolic potential encrypted in this genomic reservoir is considered to be mostly unknown and therefore a source of “genetic novelty”(Elshahed et al., 2008; Zhang et al., 2009) – which is often correlated with the concept of “microbial dark matter” due to the usual lack of cultivability of rare prokaryotes (Lynch and Neufeld, 2015; Ramond et al., 2015), therefore representing an important component of phylogenetic diversity (Lloyd et al., 2018). Notwithstanding, the rare biosphere also includes a wealth of known and culturable taxa (Shade et al., 2012; Hardoim et al., 2014; Karimi et al., 2019) that can be straightforwardly examined in biotechnology-driven research. Several recent studies suggest that the prokaryotic rare biosphere can mediate the response of natural ecosystems to environmental perturbations and pollution (Table 1). The sections below highlight metabolic, genotypic and physiological traits of reportedly low-abundance prokaryotes which illustrate possible links with biotechnology.

**TABLE 1:** Selected microbial rare biosphere studies relevant for bioremediation.

**Sample type**	**Main methods**	**Relevance**	**Bioremediation potential**	**References**
Lakewater microcosms	Serial dilution of microbial community from lake water to simulate rare species loss; Media with phenol or humic substances; Diversity assessed by T-RFLP^1^ of 16S rRNA genes; PCR-screening for the *xilE* gene.	Microbial resistance to phenol and humic substances decreases with rare species loss.	Degradation of phenol (associated with the presence of *xilE genes*, for catechol 2,3 dioxygenase).	Szabó et al., 2007
Mangrove sediment and rhizospheres spiked with petroleum	Enrichment cultures assessed by PCR-DGGE,^2^ hybridization and microarrays of petroleum hydrocarbon (PH)-degrading genes and plasmids.	Low abundance plasmids and functional genes involved in PH degradation become abundant in petroleum contaminated soils. Rhizospheres of different plant species possess their own unique community of PH degraders.	Degradation of PHs by rare biosphere members.	Gomes et al., 2010
Peatland soil	16S rRNA gene DNA-SIP, with and without sulfate.	*Desulfosporosinus* (0.006% abundance) significantly contributes to sulfate reduction.	Sulfate reduction lowers methane emission from peatland soils.	Pester et al., 2010
Marine	Continuous seawater cultures exposed to different salinity and DOC gradients; PCR-DGGE and amplicon sequencing of 16S rRNA genes.	Rare members of the community can grow abundant after disturbance and contribute to overall community stability.	Resilience toward salinity and DOC gradients.	Sjöstedt et al., 2012
Soil microcosms incubated with maize litter as alkane source	Enrichment of alkane-degrading bacteria by liquid sub-cultivation; Alkane degraders identified by *alkB* gene detection; Community diversity assessed by T-RFLP of 16S rRNA genes.	Description of previously unknown and rare alkane degraders using complementary methodologies.	Identification of a possible seed bank of rare prokaryotes able to degrade alkanes.	Giebler et al., 2013
Marine	DNA-SIP coupled with amplicon sequencing of 16S rRNA genes.	Identification of disproportionately active degraders of phenanthrene in the rare biosphere; Identification of different rare biosphere groups tolerant to PAHs in general.	Phenanthrene degradation.	Sauret et al., 2014
Diesel-spiked soil microcosm	Amplicon sequencing of 16S rRNA genes.	Identification of conditionally rare taxa that respond to perturbance.	Hydrocarbon degradation.	Fuentes et al., 2016
PAH-contaminated soil	Microfluidic spread plating (High throughput cultivation).	Methodology enables better coverage of rare prokaryotes. Identification of a rare *Blastococcus* sp. able to degrade fluoranthene.	Fluoranthene degradation by a cultured representative of the rare biosphere.	Jiang et al., 2016
Lakewater mesocosm	HPLC; Amplicon sequencing of 16S rRNA genes; Total DNA sequencing.	Rare biosphere members can respond to pollutants that are rare or absent in the environment; The response can be through growth or by HGT of plasmids with the needed degradation pathways.	2,4-dichlorophenoxyacetic acid, 4-nitrophenol, and caffeine degradation.	Wang et al., 2017
Wood log in aquaria with seawater	Amplicon sequencing of 16S rRNA genes; Sulfide detection.	“Ultra-rare” microbes can also respond to environmental shifts; functional redundancy observed in the rare biosphere.	Community functions (e.g., sulfur cycling pathways) are compromised under a certain threshold of rare species removal.	Kalenitchenko et al., 2018
Anoxic sludge	Amplicon sequencing of 16S rRNA genes; Metatranscriptomics; Sterols identification.	Identification of new cholesterol degraders in the rare biosphere (through the 2,3-seco pathway)	Anaerobic cholesterol degradation.	Wei et al., 2018
Arctic and Antarctic coastal waters	Total DNA and cDNA shotgun sequencing.	Rare biosphere members can respond to anthropogenic dissolved organic carbon (ADOC) perturbation and includes previously recognized semi-volatile organic pollutant degraders.	Hydrophobic ADOC degradation.	Cerro-Gálvez et al., 2019
Marine sponge	Bacterial cultivation with oligotrophic medium; Comparative genomics.	Low abundance, sponge-associated *Alphaproteobacteria* possess genes for heavy-metal resistance and degradation of aromatic compounds, which are characteristic of dominant yet unculturable sponge symbionts.	Bioremediation potential revealed for rare biosphere phylotypes cultivated in the laboratory. Functional redundancy hypothesis suggested to confer homeostasis to sponge symbiont communities.	Karimi et al., 2019

*^1^Terminal-restriction fragment length polymorphism. ^2^Polymerase chain reaction-denaturing gradient gel electrophoresis.*

## The Ecophysiology of the Prokaryotic Rare Biosphere

**FIGURE 1:**
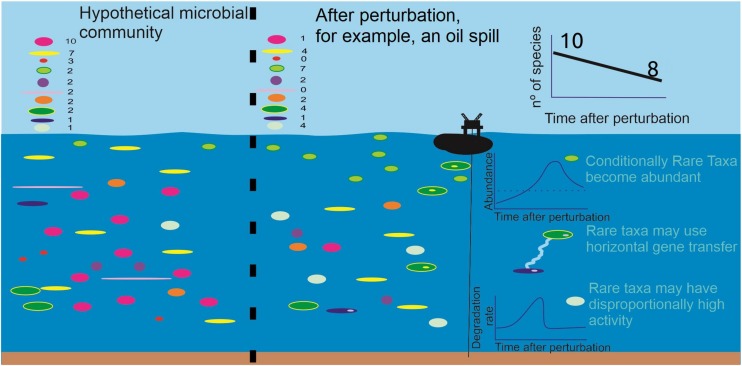
Hypothetical, oversimplified marine microbial community composed of 10 different species, originally with 32 total individuals (a proxy for e.g., 3.2 × 10^6^ cells⋅mL^−1^). Most of the species are rare and after a perturbation, such as an oil spill exemplified in the picture, it is expected that the overall diversity decreases, and that some rare species become abundant, some tolerate the perturbation and others do not tolerate the perturbation, entering local extinction. We summarize how the prokaryotic rare biosphere is thought to respond to such perturbations. Conditionally rare taxa can grow abundantly and degrade the pollutant(s) as part of their normal metabolism. When the stressor is completely degraded, they may go back to low abundance. Rare taxa, in these conditions, may also transfer functional genes to other more abundant bacteria, helping the community to cope with the perturbation. Finally, it is also thought that some rare taxa may display high activity, relative to their abundance, for the metabolism of specific compounds; they could possibly show a high degradation rate while the stressor is present, and thereafter return to low activity.

Currently, the consensus is that natural prokaryotic communities can respond to perturbations and environmental shifts through the rare biosphere (Figure 1) because of its high diversity (Jousset et al., 2017). Such a response can be explained from the perspective of the seed bank theory (Pedrós-Alió, 2006). For example, rare bacteria were found to become abundant when exposed to salinity and Dissolved Organic Carbon (DOC) gradients in seawater, while the overall functions of the community were maintained (Sjöstedt et al., 2012; Table 1). A similar study tested the effects of changing salinity and temperature on sulfide-rich spring communities, also confirming that rare prokaryotes can become abundant in response to both slow and fast perturbations (Coveley et al., 2015). Here, unique rare phylotypes were most responsive, suggesting that additional phylogenetic diversity equips the community with the ability to cope with diverse environmental changes (Table 1).

In the environment, the prokaryotic rare biosphere response is thought to be composed of at least two main mechanisms: clonal amplification, i.e., conditionally rare taxa (CRT) that become abundant with changing conditions; and/or horizontal gene transfer (HGT) of functional traits through different community members (Wang et al., 2017). Thus, CRT in the environment would respond to natural and man-made selective pressures as microbial species subjected to selective enrichments do under controlled conditions in the laboratory. This parallel between the rather recent rare biosphere and the well-established bioremediation literatures can be drawn by studies that simultaneously addressed prokaryotic community structures in the wild and in the corresponding enrichment cultures obtained after exposure to a stressor/carbon source. For example, Gomes et al. (2010) used a suite of molecular tools to examine Petroleum Hydrocarbon (PH)-degrading enrichment cultures from mangrove rhizospheres and sediments, and found that the bacterial populations, functional genes and plasmids responsible for PH degradation in the enriched cultures were below the detection limit in the source environment.

Besides CRT, some rare prokaryotic populations in the environment appear to remain active, or even increase their activity, while maintaining low or near-zero growth rates (Pester et al., 2010; Hausmann et al., 2016, 2019; Table 1). Using Stable Isotope Probing (SIP) of 16S rRNA and dissimilatory (bi)sulfate reductase (dsrAB) genes, Pester et al. (2010) revealed that *Desulfosporosinus* spp., despite representing only 0.006% of the total microbial community, significantly contributed to sulfate reduction in peatland soils. Mesocosm experiments performed with periodic supplementation of individual fermentation products (formate, acetate, propionate, lactate or butyrate) in the presence or absence of sulfate further demonstrated that sulfate turnover in peatland soils was primarily mediated by rare biosphere prokaryotes, involving both novel and already known sulfate reducing species (Hausmann et al., 2016). Among the latter, *Desulfosporosinus* spp. was considered not to grow abundant while maintaining a steadily active metabolism for 50 days, as indicated by ribosome/genome copy ratios estimated using qPCR (Hausmann et al., 2016). Finally, Hausmann et al. (2019) coupled genome-resolved metagenomics to metatranscriptomics to further explore the ecophysiology of sulfate reducing bacteria in the abovementioned mesocosms, revealing that the proposed novel species Candidatus *Desulfosporosinus infrequens* was able to concert near zero growth at low abundances with estimated high activity. This capacity was considered to result from over expression of genes for ribosome production, energy metabolism and response to stressors while displaying low expression of growth-associated genes (Hausmann et al., 2019).

This knowledge (at the genetic and functional levels) can prove useful in the industrial context because uncoupling bioproduct formation from cell growth (through proper exploitation of strains active at near zero growth states, for instance) is deemed relevant in process optimization (Ercan et al., 2015). For example, Lactic Acid Bacteria (LAB) in retentostat cultures remain viable for long periods of time, without biomass growth, after reaching the exponential growth phase (Ercan et al., 2013). This is typical in food fermentation processes, e.g., in cheese (Smit et al., 2005) and dry sausage (Hugas and Monfort, 1997) ripening, whereby LAB can undergo long periods of very low nutrient availability while, regardless, playing a role in flavor development (Hugas and Monfort, 1997; see Ercan et al., 2013 for an extended discussion).

In fact, many biotechnology studies indeed focus on metabolic engineering of well-known strains to divert carbon flow toward specific bioproducts, instead of biomass (Papagianni, 2012). Likewise, synthetic biology approaches hold promise in the development of engineered strains displaying increased tolerance to a range of stressors, thereby facilitating bioproduct formation (Jia et al., 2014). Ecologists interested in the prokaryotic rare biosphere aim to understand how prokaryotes remain viable in a non-dormant state for long periods of time (e.g., Hausmann et al., 2019), whereas biotechnology-oriented research aims at optimizing biological processes. The interface between the two research fields lies in the study of metabolic trade-offs dictating energy allocation to cell growth and/or activity, and the environmental variables/laboratory conditions that are relevant to fine-tune cellular metabolism.

## The Prokaryotic Rare Biosphere Responds to Pollutants: Potential for Bioremediation

Well known functions involved in environmental recovery from pollutants can also be stored in the prokaryotic seed bank, until they are necessary, as suggested by a lake water mesocosms experiment (Wang et al., 2017) testing the effect of different organic compounds (2,4–dichlorophenoxyacetic acid, a herbicide, 1,3,7–trimethyluric acid, caffeine and 4–nitrophenol, a pesticide) on prokaryotic community composition. Even though these compounds were not detected in the lake, rare bacteria had the genetic machinery to respond to the stressors (Table 1). Further analysis showed that several rare taxa were enriched after addition of 2,4–dichlorophenoxyacetic acid, namely *Burkholderia*, *Sphingopyxis*, and *Variovorax* spp. The genetic pathways for the degradation of the stressors were below the detection limits prior to incubation but were afterward identified, with variations across replicates (Wang et al., 2017; Table 1). Further, the catabolism of cholesterol was found to be mediated through the 2,3-seco pathway by rare bacteria from the denitrifying sludge of a wastewater treatment plant (Wei et al., 2018; Table 1). Finally, community-level resistance and degradation of phenols, which are toxic compounds often released into the environment due to industrial activities (Duan et al., 2018), has been as well related with the prokaryotic rare biosphere (Szabó et al., 2007; Table 1).

Although bioremediation procedures already exist for the treatment of oil spills, improvements are needed to minimize environmental consequences of large spill incidents (Ron and Rosenberg, 2014). It is now known that the rare prokaryotic biosphere contributes to the degradation of Polycyclic Aromatic Hydrocarbons (PAHs) in natural environments (Table 1). For instance, Sauret et al. (2014) described the *in situ* enrichment of rare biosphere populations after coastal seawater samples had been amended with phenanthrene, with both well-known (e.g., C*ycloclasticus* spp.) and then-unrecognized (e.g., *Oceanibaculum* and *Sneathiella* spp.) phenanthrene-degrading populations observed to behave as CRT under the experimental circumstances. Currently, the metabolism of known PAH-degrading bacteria is mostly studied in the laboratory and application of this knowledge to mitigate environmental pollution should be supported by *in situ* based studies (Sauret et al., 2014). In soil, while testing bioremediation and bioaugmentation strategies for oil spills, it was reported that rare microbes mediated the response to drastic stress (high concentrations of oil), and that bioaugmentation introduced novel CRT into the system (Fuentes et al., 2016). In another study, Giebler et al. (2013) identified a possible seed bank of rare prokaryotes, originated from pristine soils, with the ability to degrade alkanes. Finally, manipulative experiments with Arctic and Antarctic microplankton communities showed that the addition of hydrophobic, anthropogenic dissolved organic carbon reduced overall microbial diversity and that the degradation response was mediated by rare prokaryotes (Cerro-Gálvez et al., 2019).

Altogether, recent research suggests that low abundance prokaryotes confer resilience to natural microbial communities upon exposure to pollutants. The described responses, however, most often involved the increase in abundance of CRT present in the samples, analogously to enrichment culture experiments performed in the laboratory.

## Genetic Diversity and Novelty – Bioprospecting Potential

The pool of microorganisms currently uncultivatable in the laboratory (“microbial dark matter”), encompasses much phylogenetic novelty, including phylotypes displaying an uncommon biology with alternative metabolic pathways such as different genetic codes and unusual ribosomal composition, reflecting genes and functions that are yet to be discovered (Wu et al., 2011; Lynch et al., 2012; Rinke et al., 2013; Brown et al., 2015; Solden et al., 2016). The prokaryotic rare biosphere may include groups that are phylogenetically close or distant from abundant taxa (Elshahed et al., 2008), also identified among rare eukaryotes (Logares et al., 2014; Debroas et al., 2015). Phylogenetically distant prokaryotic taxa were found to contribute more to community turnover after new perturbations (Coveley et al., 2015), suggesting phylogenetic diversity in the response process. In sulfur springs, an approach combining high throughput and Sanger sequencing technologies revealed that several rare, unclassified lineages did represent novel phyla and classes (Youssef et al., 2012). Similar findings were reported for Arctic tundra soils, where a component of the rare biosphere included previously unknown taxa (Lynch et al., 2012).

Many unknown functional genes from the rare biosphere might yet be inaccessible, even with the current power of high throughput sequencing techniques (Ekkers et al., 2012; Lynch et al., 2012; Crespo et al., 2016). Moreover, the fact that a large portion of this community is hitherto uncultivatable remains one major hindrance in bioprospecting for novel activities within the unknown component of the rare biosphere. To effectively exploit such a vast reservoir of prokaryotic diversity, blending of multiple, advanced technologies is needed to further our knowledge of the prokaryotic rare biosphere beyond mere rRNA gene sequencing.

Continued investment in total community, metagenomic DNA sequencing coupled to the subsequent binning of MAGs from diverse environments is expected to substantially improve knowledge of the coding potential (Brown et al., 2015) and bioremediation and biogeochemical cycling (Hausmann et al., 2019) functions within the rare biosphere in the following years. However, there is currently a gap between the number of “new” proteins predicted from genome annotations and those which are fully characterized (Galperin and Koonin, 2010; Bastard et al., 2014). Improving genome annotations through experimental characterization of novel proteins will be paramount to increase our knowledge of the functional attributes of rare prokaryotes. Alternative cultivation methodologies, especially when complemented by genome sequencing (“culturomics”), can increase our ability to document the diversity and function of low-abundance prokaryotes and eukaryotes, including fungi (Lagier et al., 2012).

Comprehensive culturing, either involving taxon-specific or oligotrophic medium compositions, often permits access to diverse, rare prokaryotes not depicted by molecular techniques alone (Hardoim et al., 2014; Zehavi et al., 2018; Karimi et al., 2019; Rego et al., 2019) while enabling access to their metabolism and bioactivities. For instance, broad-spectrum antimicrobial activities have been reported for freshwater sponge-associated *Pseudomonas* spp. (Keller-Costa et al., 2014) which did not rank among the dominant bacteria in the system (Costa et al., 2013). Currently, access to much novel secondary metabolism among prokaryotes is being achieved by combining genomics, computational biology and analytical chemistry to the study of rare or “hard-to-culture” bacteria (Helfrich et al., 2019; Silva et al., 2019). Finally, the use microfluidics in high-throughput cultivation enables the screening for bioremediation functions among rare taxa in a complex community (Jiang et al., 2016; Table 1).

## Concluding Remarks

A significant component of the known and unknown prokaryotic diversity exists at low abundance in the environment. This mini review bridged recently developed concepts regarding the ecophysiology of low abundance prokaryotes with current knowledge from biotechnology. Many CRT have been reported to respond to pollutants in the environment, whereby the active response was not necessarily performed by well-established strains already in use for bioremediation processes. Furthermore, most low abundance prokaryotes remain uncultured and consequently understudied, being an important source for bioprospecting new functions, as well as gene and protein microheterogeneities underlying the expression of already known functions. Although very useful in biodiscovery, metagenomics-centered methods alone will not suffice to truly illuminate the breadth of potential new functions or variations of known functions hidden in the prokaryotic rare biosphere, neither improve their use in applied biotechnology. To this end, coupling culturomics to DNA mutagenesis research will be ultimately necessary to assign novel functions to the wealth of so-far hypothetical proteins which still dominate genome annotations of even the most well-known and studied prokaryotes. Moreover, recent advances in genome editing technologies and synthetic biology hold much promise in leveraging our capacity to engineer e.g., pollutant-removing (Dvořák et al., 2017), drug-producing (Wang et al., 2019) and stress-tolerant (Jia et al., 2014) bacteria, thus facilitating our ability to harness the metabolism of both culturable and thus far unculturable low abundance prokaryotes in biotechnology.

## Author Contributions

FP, CM, and RC conceptualized the manuscript. FP wrote the main manuscript. CM and RC reviewed and improved the manuscript.

## Conflict of Interest

The authors declare that the research was conducted in the absence of any commercial or financial relationships that could be construed as a potential conflict of interest.
